# Hospital managers’ views on the state of patient safety culture across three regions in Ghana

**DOI:** 10.1186/s12913-022-08701-z

**Published:** 2022-10-29

**Authors:** Immaculate Sabelile Tenza, Priscilla Y. A. Attafuah, Patience Abor, Edward Nketiah-Amponsah, Aaron Asibi Abuosi

**Affiliations:** 1 School of Nursing Science, Faculty of Health Sciences, North-West University, Potchefstroom Campus, Potchefstroom, South Africa; 2 South African Research Chairs Initiative (SARChI), School of Public Health, Faculty of Health Sciences, University of the Witwatersrand, Johannesburg, South Africa; 3 Public Health Nursing Department, School of Nursing and Midwifery, University of Ghana, Legon, Ghana; 4 Department of Public Administration and Health Services Management, University of Ghana Business School, Legon, Ghana; 5 Department of Economics, School of Social Sciences, University of Ghana, Legon, Ghana

**Keywords:** Patient safety culture, Hospital managers, Ghana

## Abstract

**Background:**

Improving patient safety culture in healthcare organisations contributes positively to the quality of care and patients’ attitudes toward care. While hospital managers undoubtedly play critical roles in creating a patient safety culture, in Ghana, qualitative studies focussing on hospital managers’ views on the state of patient safety culture in their hospitals remain scanty.

**Objective:**

This study aimed to explore the views of hospital managers regarding compliance to patient safety culture dimensions in the selected hospitals in the Bono, Greater Accra, and Upper East regions of Ghana.

**Methodology:**

This was a qualitative exploratory study. A purposive sampling of all hospital managers involved in patient safety practices was conducted. The sampled managers were then invited to a focus group discussion. Twelve focus group discussions with each consisting of a maximum of twelve participants were conducted. The ten patient safety culture dimensions adapted from the Agency for Healthcare Research and Quality’s patient safety culture composite measures framed the interview guide. Deductive thematic content analysis was done. Lincoln and Guba’s methods of trustworthiness were applied to ensure that the findings are valid and reliable.

**Findings:**

Positive patient safety culture behaviours such as open communication, organisational learning, and strong teamwork within units, were an established practice in the selected facilities across Ghana. Lack of teamwork across units, fear of reporting adverse events, the existence of a blame culture, inconsistent response to errors, extreme shortage of staff, sub-standard handover, lack of management support with resources constrained the patient safety culture. The lack of standardised policies on reporting adverse events and response to errors encouraged managers to use various approaches, some resulting in a blame culture. Staff shortage contributed to poor quality of safety practices including poor handover which was also influenced by lateness to duty.

**Conclusion:**

Prompt and appropriate responses by managers to medical errors require improvements in staffing and material resources as well as the enactment of standard policies across health facilities in the country. By so doing, hospital managers would contribute significantly to patient safety, and help build a patient safety culture in the selected hospitals.

**Supplementary Information:**

The online version contains supplementary material available at 10.1186/s12913-022-08701-z.

## Background

Worldwide, patient safety is of grave concern, this is because avoidable adverse events, errors and risks associated with health care remain a major challenge [1]. According to the World Health Organization (WHO), patient safety refers to the prevention of errors and adverse effects associated with health care [2]. Empirical evidence suggests that one of the key contributors to patient safety is the creation of a patient safety culture in a healthcare organisation [3–6]. Patient safety culture is defined as values, habits, and beliefs about how things operate in healthcare organisations, resulting in behavioural norms that promote safety [7]. In essence, experts in the field of patient safety culture have agreed on certain characteristics for assessment in health institutions. These characteristics are sometimes referred to as dimensions [8–12]. In this study, we referred to these characteristics as the dimensions of patient safety culture as adapted from the Agency for Healthcare Research and Quality (AHRQ)‘s ten patient safety culture composite measures [8]. The terms dimensions and composite measures as suggested by Sorra et al. [8], can be used interchangeably. These include communication about errors; communication openness; handoffs and information exchange; hospital management support for patient safety; organizational learning; reporting patient safety events; response to error; staffing and work pace; supervisor, manager, or clinical leader support for patient safety; and teamwork [8].

There is also increasing recognition of the important role played by hospital leaders or managers in inculcating patient safety culture in healthcare organisations [13]. Although there is a thin line between leaders and managers, in this study, the managers (consisted of departmental or unit heads) and refer to all the middle and front-line level managers who took roles as unit or departmental heads or in an acting position in the selected hospitals of the three regions in Ghana. This study was interested in their views on the compliance to the dimensions of patient safety culture in their hospitals. This is because as frontline supervisors and managers, their evaluation of patient safety culture provides insight into the context of relevant recommendations and serves as a critical step to outline required future projects aimed at improving quality and patient safety [13]. Since the patient safety culture dimensions of AHRQ assesses both management and supervisor support for patient safety culture, in our study, management support referred to the support of the highest level such as chief executive and directors of the hospitals. These individuals were not selected as participants for the study. Supervisor support referred to the support given to the participants by their immediate supervisors.

Globally, the notion of patient safety culture remains topical amongst patient safety scientists [14]. Although patient safety culture tends to be a context-specific phenomenon [10], there are also many similarities in challenges and successes across different countries. Such similarities provide an opportunity to learn from other countries’ successes and challenges. Studies on patient safety culture have mostly found teamwork, punitive response to error and management support for patient safety to be rated high by participants [15–17]. Additionally, patient safety studies have included views from various categories of health professionals, including managers, physicians, registered nurses, and enrolled nurses [15–17]. This is because patient safety should be everybody’s business in a hospital setting.

Notably, patient safety culture studies have followed various methodologies, including systematic reviews, qualitative methods, and quantitative methods [18–20]. A 20-year-long scoping review on the type and prevalence of patient safety culture methodologies, including 107 studies, found that the quantitative approaches are dominating, and often use self-administered quantitative surveys [21]. Likewise, a 2021 systematic review of methodologies used to investigate patient safety culture dimensions including 694 studies, found that only 31 studies were qualitative. In both studies [9, 21] authors raised concerns over the dearth of qualitative studies investigating patient safety culture and advocated a need to utilise qualitative approaches to understand the detailed issues contributing to delays in achieving positive patient safety culture.

In Ghana, the Ministry of Health developed and launched Ghana’s National Healthcare Quality Strategy [22] however, the success of the implementation of such a strategy depends on the culture in the health establishments [23–25]. Patient safety studies in Ghana have found that teamwork and organisational learning are viewed as important dimensions to achieving patient safety culture [26]. While one study [5] found that management support had a significant relationship with patient safety culture in a teaching hospital in Ghana, a 2020 study in a teaching hospital found an extremely low rating on overall patient safety culture [5]. Similarly, Akologo, [27] also found a non-punitive response to error, rated very low in the hospitals of the Upper East region of Ghana.

These studies were mostly quantitative and included front-line healthcare providers. We note that in Ghana, there remains a gap in studies that explore the current practices regarding patient safety culture. More specifically, the dominating quantitative studies have not probed the reasons behind the low and high ratings to understand the current failure to comply with the desired patient safety culture. Qualitative approaches to the investigation of patient safety culture could allow in-depth clarity of the gaps in practice, devise context-specific recommendations and improve the patient safety culture. Since hospital managers have a vested interest in monitoring patient safety practices, this study explored their views regarding compliance to patient safety culture dimensions in their hospitals. The study contributes to the international discourse on possible contributors to compliance or lack of compliance with patient safety culture dimensions.

## Methods

### Study setting

This study was conducted in the three ecological zones of Ghana (i.e., the southern zone, middle zone, and northern zone). The northern zone consists of five regions in the northern part of the country while the middle zone is made up of seven regions located in the middle part of the country. The southern zone is made up of four regions along Ghana’s coastline. One region was selected from each zone, based on proximity to the authors, for example, the Bono region for the middle zone, the Greater Accra region for the southern zone and the Upper East region for the northern zone. Within each selected region, four hospitals were selected, including a public hospital, a faith-based hospital, a private hospital, and a regional hospital. There is usually more than one private health facility in the region. For example, in the Bono region, there were two other private hospitals but hospital attendance for the selected facilities was higher in comparison. Most regions have only one public and faith-based hospital so there were no other options. There is only one regional hospital in each of the selected regions. The bed capacity of the health facility, as well as the availability of the facility to consent to participate, were criteria for selection in cases where there were more than one health facility.

The organisational structure of most Ghanaian hospitals differs along the various levels of care. In the regional and tertiary hospitals, these are similar. There is a high level of hospital management which is headed by the Medical Director and supported by the Heads of the Directorates of the Hospital, i.e., Heads of Clinical Care, Nursing Services, Pharmaceutical Services, Health Administration & Support Services (HASS) and Finance & Accounts Directorate. These heads of directorates have departmental or unit heads who work under them. In the lower levels of care, there is a medical superintendent in the public hospitals who is also called the medical director in the private and faith-based health facilities. In private facilities, the medical director is usually the owner of the facility hence final decision-making rest on him. They also have departmental heads instead of directorates. The managers who consisted of departmental or unit heads were the participants interviewed in this study.

### Study design

A qualitative study design was chosen to explore the views of hospital managers on their hospital’s compliance to patient safety culture dimensions.

### Participant selection

A purposive sampling of hospital frontline and middle managers was done to ensure that the selected participants have rich information relevant to the study. All heads of clinical and non-clinical units were included in the study, and all other staff members who were not in a leadership role were excluded. The clinical hospital managers include nurse managers, pharmacy managers, laboratory heads, and administrators. The non-clinical units were those at the account’s office, janitorial heads, quality assurance managers, and administrator managers. These people in one way or the other have a bearing on patient safety. For example, the accounts manager oversees the disbursement of money for purchasing resources and equipment needed in caring for patients. Janitorial heads are responsible for ensuring a well-cleaned environment and no spills on floors to prevent no slippery floors to prevent falls as well as minimize nosocomial infections. The sampled hospital managers had at least 1 year of experience in leadership positions and were willing to participate in the study. In all, twelve [12] Focus Group discussions (FGD) were held each with a maximum of 12 participants. A total of 114 participants were involved (Additional file 1: Table S1).

### Data collection instrument

We developed an interview guide in English, which is the official business language in Ghana. The interview guide consisted of semi-structured questions, informed by the ten dimensions of patient safety culture adapted from AHRQ [8] and the research objective. The overall guiding question was “To what extent are the patient safety culture dimensions practised in this hospital? Then each of the dimensions had questions focused on it, and on how it is practised in the institution. The dimensions of patient safety that were included in the interview guide were: communication about errors; communication openness; handoffs and information exchange; hospital management support for patient safety; organizational learning; reporting patient safety events; response to errors; staffing and work pace; supervisor, manager, or clinical leader support for patient safety; and teamwork [8] (Additional file 2: Table S2).

The interview guide consisted of some probes, to ensure the participants responded based on their real observations not on what they wished should be happening.

Following the completion of designing an interview guide, a team of patient safety and quality researchers reviewed it for content validity and clarity of questions. The guide was further piloted with one focus group in a region which was not to be part of the study, and there were no substantive changes necessary after the pilot.

### Data collection

The data collection was conducted by a team of six researchers who are trained in focus group interviews with prior experience in qualitative data collection. They visited the facilities in pairs, one with the role of interviewing, the other with the role of notes taking and managing the recording. In preparation for the interviews, participants were contacted via email to request voluntary participation in the study. Following informed consent, the interviewer arranged the interview date and time with the participants with the permission of the hospital managers.

All interviews were conducted in English, in an enclosed space provided by the hospital to ensure privacy and minimize distractions. The interview began with an introduction to the study, and an explanation of the voluntary nature of participation. Participants were asked to sign a confidentiality agreement to protect all the information shared in the discussion and to put them at ease as they discuss their views. Following informed consent, the interviewer used the semi-structured interview guide to explore the views of hospital managers on the extent to which patient safety culture dimensions are adhered to in their hospital. Each interview lasted for about an hour, although the duration varied depending on the responses provided by the participants.

Interviews were recorded digitally and labelled with an FGD code. Immediately after the interviews, the interviewer wrote a synopsis of each interview to support data analysis. All audio recordings are kept on a password-protected computer to ensure confidentiality, and only the research team has access to the data.

### Data analysis

The interviews were transcribed verbatim. One research assistant who was also part of data collection (DN) cleaned the data by reading each transcript while listening to the original recording. Following data cleaning, two researchers (IST & PYAA) analysed the data independently to achieve intercoder agreement, one a health systems researcher with expertise in qualitative data analysis, and the other a patient safety and quality researcher. We agreed on following deductive thematic approach [28] to analyse the data. Codes were done independently by each researcher and one researcher who was involved in data collection, read through the codes to validate if they are a true reflection of the interview discussions. We held coding meetings to discuss and agree on codes as they emerged in each region. Both data analysts analysed the emerging codes independently for recurring patterns of meaning, and contradictions within and between transcripts, and used participants’ words from the codes to develop sub-themes. Another meeting was held to agree on the finalised sub-themes. The data analysts further interrogated and evaluated themes for similarities and differences in meaning across different regions, and to separate which of the dimensions are viewed as complied to, and those that require improvement. The finalised themes were then sent to the rest of the team to validate if they are a true reflection of the data as emerged during the interviews.

### Trustworthiness and rigour

We applied Lincoln and Guba’s [29] criteria of trustworthiness in the study. The participation of two researchers in data analysis ensures the reliability of the findings. Attaching excerpts of narratives in the report writing to illustrate themes ensures confirmability. The iterative process of repetitive listening to the audio records during data cleaning allowed for prolonged engagement with the data, thereby ensuring credibility. We read the synopses of interviews and used these as a reference to validate codes during the analysis of the rest of the data and to confirm the final generated themes.

### Findings

A total of 114 participants agreed to participate in the FGD interviews, and there was only one refusal with no specified reasons. The participants consisted of various hospital managers, such as Nurse managers (*n* = 70), Pharmacy managers (*n* = 10), Accountant managers (*n* = 12), Janitorial heads (*n* = 2), Quality assurance managers (*n* = 4) Laboratory heads (*n* = 6), Administrator managers (*n* = 10), (Additional file 1: Table S1).

Table 1 indicates the themes and subthemes that emerged.

**Table 1:** Emerging themes and subthemes

Themes	Sub-themes
Communication about error	• Various communication approaches
Communication openness	• Easy communication
Handoffs and information exchange	• Good, detailed handover • Sub-standard handover
Hospital management support for patient Safety	• Failure to create general safety in hospital • Reactive management approach to safety problems
Organizational learning	• Planned and structured organisational learning • Unplanned and unstructured organisational learning • Lack of implementation of knowledge
Reporting of patient safety events	• No structured approach to reporting • Selective and non-selective reporting of errors • Fear of reporting
Response to error	• Unstandardised approaches in responding to error • The nature of an error influences the response • Blame culture still exists
Staffing and work pace	• Contributors to staff shortage o Shortage of permanent staff & ageing workforce o Problems in staff recruitment o Poor management of leave & placement of ill staff • Consequences of staff shortage o Staff shortage compromised safety
Manager/Supervisor support for patient safety	• Consultation with frontline personnel • Lack of support with resources
Teamwork	• Teamwork within units • Lack of teamwork across units

### Communication about an error

Participants believed that there was generally good communication amongst colleagues but reserved communication with seniors.

#### Various communication approaches

There were reported diverse communication approaches across all regions, such as the use of formal handover, meetings, conferences, and WhatsApp messages were also becoming commonly used. Moreover, participants in all regions expressed positive views of communication openness in their units, and this manifested in the ease of communication amongst themselves and to their supervisors if they see something that may negatively affect a patient. The following excerpts underscore the existence of communication about errors.

*“Due to COVID-19, there were issues with holding meetings. … a WhatsApp group so that if there’s any information … , we can put it there for staff to have access to. … and as in-charges of the unit or department heads too, we have also formed a WhatsApp group because of COVID-19.” (Upper East region, Bongo, Participant 1)*

*“I get to talk to each one of us. When we are handing over, we also put it on our WhatsApp platform” (Bono region, Holy family hospital, Participant 37)*

*“Besides the monthly ward conferences, we also hold emergency meetings so when there is a mistake, we hold an emergency meeting and then we discuss it there and then we correct ourselves immediately.” (Greater Accra region, Amasaman hospital, Participant 63)*

### Communication openness

Participants in all regions expressed positive views of communication openness in their units, with an emphasis that staff spoke up freely amongst themselves and to their supervisors if they see something that may negatively affect a patient.

#### Easy communication

When reflecting on easy communication, participants had this to say:

“*At times they discuss patient safety issues amongst themselves, and at times it comes to me and we all discuss it together, ...” (Bono region, Bono regional hospital, Participant 38)*

*“Okay, so they are very free to talk. …, approach me and we will discuss it together so that’s what we’ve been doing.” (Greater Accra region, Amasaman hospital, Participant 66)*

*“I think in this hospital communication has been fantastic from management to the lower level. They are approachable you can just go to them, so the response too sometimes is good…” (Upper East region, Bongo hospital, Participant 27)*

### Handoffs and information exchange

Some participants from Bono and Greater Accra regions believed that there was a good and detailed confidential handover and information exchange in their hospitals, while there were concerns from the Upper East region that the standard of handover had deteriorated. Lack of accompaniment of patients to the wards, and staff reporting late to work were major contributors to poor handover and patient safety gaps.

#### Well detailed handover

Participants were of the view that a detailed handing over is very necessary therefore they do not accept patients transferred with inadequate information.

*“… if you come to the ward without the adequate information, you are usually sent back to the unit to go and take the right information before you bring the client ...” (Greater Accra region, Pentecost hospital, Participant 65)*

#### Sub-standard reporting and handover

Lack of patient accompaniment, shortage of staff and lateness to duty, were the most cited reasons contributing to poor handover practices.

*“Handing over is a major challenge within the ward and also from department to department and sometimes they attribute it to the staffing issue. … the OPD instead of a nurse accompanying the patient they sometimes give to a student …, there are communication gaps …” (Upper East region, Bolga hospital, Participant 5)*

*“On the issue of handing over from ward-to-ward, we have a very big problem with the OPD, sometimes they send a patient alone.” (Upper East region, Bolga hospital, Participant 7)*

*“When you are handing over and a particular staff member is not in the ward at that time (due to late coming), handing over is done but he or she may not be able to know what is happening at that time.” (Upper East region, Bawku hospital, Participant 9)*

### Hospital management support for patient safety

Hospital management referred to the highest level of decision-makers of health facilities. Support by these executive members for patient safety was discussed here. There were general concerns about the lack of management support with resources. When reflecting on hospital management’s support, failure to create safety in the hospital and reactive response to safety took a centre stage in the discussions.

#### Failure to create general safety in hospital

With regard to the failure to create general safety in hospitals, participants expressed concerns about hospital managements’ lack of urgency to create safety in the hospital, although this is not an integral part of patient safety care processes. Participants were not satisfied with the level of security in the wards, a clear indication of management’s lack of prioritization of same.

*“For instance, we don’t have a security guard in the wards and prioritising of security guards too is a sign that hospital management takes safety very seriously.” (Upper East region, Bongo hospital, Participant 11)*

*“Management can do more, we don’t have ward security, and sometimes we have theft cases in the ward.” (Bono region, Holy family hospital, Participant 40)*

#### Reactive management approach to safety problems

Hospital management was rather reactive to patient safety concerns. Moreover, the lack of funds for the timely acquisition of tools needed for work was identified as a hindrance to patient safety.

*“Most of the time they're always reactive, for example when we hear that accreditation is coming, NHIS accreditation, GHS Regulatory body is coming, they need this and that. Then you see that management is rushing to get all those documents ready, immediately they go, then we forget waiting for the next year …” (Upper East region, Bawku hospital, Participant 34)*

### Organizational learning

Organisational learning focused on how the hospitals learnt from mistakes and on how they evaluate changes for effectiveness. Participants across all regions took the opportunity to describe how they intentionally create an environment for learning from the mistakes of others. They achieve that through structured and unstructured approaches. For example, they held staff meetings, mortality and morbidity meetings, and monitoring and reporting feedback meetings, to reflect on the existing mistakes, and learn from them. In addition, they organised structured in-service training, and orientation of new staff, to emphasise learning from the errors and teach the expected best practices. The formal training also allows for the evaluation of changes for effectiveness, through training evaluations. Unstructured approaches included close supervision of junior staff, and random teaching of staff when they reported an error. However, there was also a pressing concern about the lack of implementation of the knowledge learnt and the lack of follow-up structures to evaluate on-going learning.

#### Planned and structured organisational learning

Participants who shared examples of planned structured organisational learning which improved performance in their institutions, had this to say:

“*We organized patient safety-related in-service training for some of our night supervisors and it was successful, and we have seen the shift in performance. We have improved in the way we used to do our night supervision and I think the current state is good.” (Upper East region, Bongo hospital, Participant 32)*

*“As a department, we have clinical meetings with a focus on patient safety, once a week so it’s on zoom. Doctors present topics then we do mortality meetings, we learn and then they take our inputs.” (Greater Accra region, Ridge hospital, Participant 70)*

#### Unplanned and unstructured organisational learning

Other participants also shared an example of random unstructured organisational learning.

*“Usually in your work, you have a senior who is experienced, who will try to correct you and encourage you to do the right thing. So, we also have internal systems where we try to correct ourselves so that we do the right thing.” (Bono region, Sampa hospital, Participant 44)*

*“If an incident occurs, normally we put it on our communication platforms just to alert some of us to be very careful of not repeating the same incidents and then during ward meetings, we talk about it.” (Bono region, Bono hospital, Participant 47)*

#### Lack of implementation of learning

Concern over lack of implementation of learning was also attributed to lack of support with resources, lack of motivation to implement new knowledge, coupled with lack of enforcement and was expressed as follows.

*“For instance, if I go for handwashing workshops, you should expect that it (knowledge and skills) must be applied. … some managers are not bothered to find out whether the knowledge from training is implemented, and that is where the issue is.” (Upper East region, Bolga hospital, Participant 2)*

### Reporting of patient safety events

Concerning reporting patient safety events, in some hospitals, there was no structured approach to reporting, for example, one participant mentioned that people report but there is no reporting book. Participants also believed that there was sometimes selective reporting of errors, depending on whether the patient is aware of the error. For example near accidents were not always reported. There was also a general sense that there was fear of reporting.

#### No structured approach to reporting

Most of the participants in the Upper East region thought that there was no culture of compulsory reporting of errors.

*“You see an incident report should have been formal, every unit is supposed to have one, but since I came here, I have not seen something like that.” (Upper East region, Bongo hospital, Participant 20)*

*“The only challenge that which I think we should be looking at is that we should put systems in place so that some of these errors could be recorded or documented so that as a team or a hospital we will be able to rectify some of these errors.” (Upper East region, Bolga hospital, Participant 24)*

#### Selective reporting and non-selective reporting of errors

Participants from various regions attested to fear of reporting, at times leading to selective reporting of errors due to fear of being stigmatised.

#### Fear of reporting

*“In response to errors, I think all staff is supposed to report but is not all staff that report. Some feel shy to report the errors, when people realize that they have committed mistakes they will see themselves as inferior.” (Upper East Ghana, Bongo hospital, Participant 23).*

#### Selective reporting

*“It depends on the error, but for critical errors, we report all critical errors.” (Bono region, Holy family hospital, Participant 46)*

*“Stigmatization. For example, if you report a needle prick after attending to a patient, some colleagues will judge you as incompetent. On the other hand, if the patient has an infectious disease like HIV/AIDS, then you have to start taking anti-retroviral medications and people start suspecting you are infected ... So, people choose which errors to report and the ones to shelve.” (Upper East region, Bongo hospital, Participant 28)*

One participant suggested that good interpersonal relationship increases the possibility to report errors, by subordinates.

*“If you have very good interpersonal skills, I’m sure people would be able to come to you and report issues but when we (*managers*) do not have good interpersonal relationships ...” (Greater Accra region, Ridge hospital, Participant 73)*

### Response to error

The participants across all regions believed that it was important to question the person who made an error before engaging with the rest of the team. However, participants verbalised that the approach to the questioning of errors, and managers’ reactions after questioning depended on the manager’s style, the nature of the error and hospital protocols. Participants concurred that the blame culture still existed and that people who made errors were questioned or punished depending on the nature of an error.

#### Approaches in response to an error

In the Upper East region, the participants highlighted three approaches to responding to errors, one emphasizing the hierarchical nature of reporting, the other emphasising politeness as an approach to achieve learning, and finally a need to investigate, resolve and teach.

*“We’ve put in mechanism, at the ward level, if you have any such incident you report to the in-charge and when the in-charge take notice of this, he draws the office of the matron’s attention. Then quickly, a team is constituted to investigate the alleged unsafe practice and then the feedback is given to the client. …” (Upper East region, Bawku hospital, Participant 31)*

An emphasis on the need to be polite while dealing with errors to encourage learning to take place was also alluded to:

*“But one thing I have observed is, most people when they make the error and you want to correct them while you are not polite, in the end there is no learning. ...” (Upper East region, Bolga hospital, Participant 21)*

An approach to investigate, resolve & teach was indicated as follows:

“*... Now when it’s reported, we investigate it and give feedback to the family. We take that as an opportunity to train or provide refresher training for staff. ...” (Upper East region, Bawku hospital, Participant 35)*

In the Greater Accra region, the participants followed the approach of verbal questioning of personnel, followed by investigation, which is usually followed by a warning, and teaching of the rest of the team.

*“So, what usually happens is that, if there is an error, the person is called to enquire why that particular mistake happened. If you explain whether verbal or written and your explanation is not understood, there can be a committee set to investigate whatever happened and either a warning will be given to you, you will be asked to sign a bond of good behaviour ...” (Greater Accra region, Pentecost hospital, Participant 79)*

#### The nature of an error influences the response

However, the participants from the Greater Accra region further pointed to the need for an immediate assessment and intervention depending on the type of error or the manager’s style.

*“I think if a staff commits an error, you have to assess the extent of the error because some of the errors have to be intervened immediately. When it comes to procedure and caregiving, some of them you have to intervene because if for instance if someone is giving an injection and you know the site is not right, immediately you have to intervene ...” (Greater Accra region, Pentecost hospital, Participant 81)*

Supervisors’ preferences also contributed to the response to errors.

*“It depends on your supervisor, others don’t mind chastising you even in front of patients and your other junior colleagues and that one if you are not careful, you might be tempted to also reply to the person.” (Greater Accra region, Amasaman hospital, Participant 83)*

#### Blame culture still exists

Participants from the Bono region followed the same approach as Greater Accra in questioning the person who committed an error, but they emphasised that they pay particular attention to the repetition of the same error by the same person, and feel that justifies a blame culture.

*“When you feel like this person he has done ABC and I have corrected him on it and yet the person is still repeating the same thing, in that case, we have to blame them.” (Bono region, Sampa hospital, Participant 50)*

Some of the participants who shared the need to blame had this to say:

*“We talk to them, but if they have repeated, they are blamed.” (Bono region, Owusu hospital, Participant 53)*

*“Some of them you have to blame for them if not next time they will do a worse thing.” (Upper East region, Bolga hospital, Participant 36)*

### Staffing and work pace

All participants across three regions believed there was generally a shortage of staff and a high workload which negatively affected work pace. There was consensus across regions that the shortage of staff contributed to emotional distress and compromised quality of care, infection control practices and patient safety. However, there were varying views on what contributes to the staff shortage in each region.

#### Shortage of permanent staff and ageing workforce

In the Upper East region, participants verbalised gaps in employment of permanent staff. Additionally, there were aged employees who are reluctant to learn new skills, thereby creating a shortage:

*“For staffing in my unit, it’s a bit challenged because the entire work of the health information unit is so demanding, and we are only two.” (Upper East region, Bongo hospital, Participant 4)*

*“Management has employed some casual workers to help us because of workload. If you look on paper at the total number of staff in the unit including the casual workers, it looks just okay. But some staff in the unit are just adding to the number....” (Upper East region, Bolga hospital, Participant 6)*

#### Problems in staff recruitment

Participants from the Bono region shared the same sentiments on staff shortage, but one distinct reason for the shortage was difficulties in recruitment.

*“A lot of people who are posted (allocated) to Sampa will be like the place is far, I don’t know the place, I won't go, they will go and try to change the posting (allocation) so I am sure our staffing issues can be improved if people accept where they have been posted (allocated).” (Bono Region, Sampa hospital, Participant 45)*

#### Poor management of leave, and placement of ill staff

Meanwhile, participants from the Greater Accra region verbalised concerns of poor management of leave, coupled with poor placement of staff major contributors to an impression of staff shortage.

*“Well, I think that the unit has adequate staff but the reason why this challenge is present is because of the artificial shortage. People are on maternity leave, study leave and sick leave …” (Greater Accra region, Ridge hospital, Participant 101)*

“*You may think you have enough (staff) but when they start going on leave, then you realize no you don’t have enough *…” (Greater Accra region, Pentecost hospital, Participant 110)

*“There are many staff who have health conditions, some even have psychiatric conditions and all others and yet they have been put in places to care for patients, so they’ll come to work in person but in actual work output, they are not helping.” ( Greater Accra region, Ridge hospital, Participant 112)*

#### Shortage of staff compromised safety

Participants further lamented that the consequences of the shortage of staff were increased workload, compromised quality of patient care and safety, exacerbated by increased stress and emotional distress on health care workers.

*“Safety is compromised, more especially in areas where the units are seriously understaffed. This is because with extreme staff shortage, the staff begin to cut corners in providing services and … patient care standards are there to ensure that the quality of service that we render is as expected ...”(Upper East region, Bawku hospital, Participant 10)*

### Leader/managerial /supervisor support for patient safety

This theme focused on the support from immediate supervisors of the participants. The participants across all regions when asked about their immediate manager’s support for patient safety thought that there was existing verbal support from the supervisors, yet they lacked action. For example, their supervisors made efforts to incorporate participants’ opinions on patient safety. However, there was a lack of support with resources; the immediate supervisors were not willing to spend or motivate the provision of resources. Most participants lamented the health system challenges, such as the lengthy procurement processes leading to delayed resources, dilapidating and unsafe infrastructure, and lack of standardised patient safety protocols.

#### Consultation with frontline personnel regarding patient safety

One participant who commented on staff involvement in patient safety issues had this to say:

*“Supervisors brought about some questionnaires to some staff so they will be able to know our opinions concerning certain areas (of patient safety). So aside the meetings, the ward meetings and forums, we have questionnaires that are given to us to help us to put our ideas on board.” (Greater Accra region, Ridge hospital, Participant 114)*

#### Lack of support with material and financial resources

Participants across all regions had a concern over the lack of support with resources leading to improvising and compromise of patient safety. Their challenges included failure to provide adequate material resources.

*“The resources that we need to work with are not there so when they know that you want this they will give few items…they are not providing adequately for us to work effectively.”( Upper East region, Bongo hospital, Participant 8)*

*“There are no bedside rails, … they will say we don’t have enough, they are waiting for something to happen ...”(Bono region, Bono hospital, Participant 34)*

There were also concerns over reluctance to spend:

*“Normally, there is no supervisor who wants to spend money anyhow, so if the problem entails procuring something to curb a particular situation, they are always reluctant.” ( Greater Accra region, Pentecost hospital, Participant 90)*

### Teamwork

Participants from the three regions believed that there is teamwork within units and that effective teamwork facilitated the achievement of their goals. Some participants from Bono and Greater Accra regions acknowledged that teamwork in their regions was facilitated by planning and clear allocation of duties. However, some participants from the Upper East region, pointed out that not everyone had internalised the importance of teamwork. They also had the opinion that the teamwork was good within the units but there was poor inter-departmental teamwork.

#### Teamwork within units

Participants who attested to the presence of teamwork had this to say:

*“You cannot work as an individual in a Covid-19 holding centre, we plan our activities for the day, we decide who goes to the patients area, who helps with the decontamination, so the teamwork is very effective in achieving our target for every day.” (Bono region, Bono, Participant 41)*

*“With general surgery, I will say teamwork is great there. … there is a division of labour so we all work towards the goal that we have set and then we yield the result.” (Greater Accra region, Ridge, Participant 93)*

Those who had the impression that their colleagues were not self-driven to teamwork had these comments:

*“For my unit, I have realized that sometimes some of the staff when they are less busy and the other side is very busy they sit without going to support others unless a superior comes and says can you go and support the others. I expect that if the teamwork is there when you see that you are less busy you should get up and see how your other colleagues are doing.” (Upper East region, Bolga, Participant 48)*

*“We still have the majority of the staff that thinks that teamwork is not encouraged even though we can boast of teamwork but there is still a gap as far as teamwork is a concern.” (Upper East region, Bolga, Participant 51)*

#### Lack of teamwork across units

One of the participants who commented on the lack of inter-departmental teamwork referred to the lack of teamwork between the pharmacy and the wards:

*“Like a prescriber sitting in his consulting room prescribing drugs that are not available at the pharmacy. This tells you there is a problem because if there is teamwork, the prescriber should be able to know the drugs that are available in the pharmacy. ... There's no teamwork between different departments.” (Upper East region, Bawku, Participant 60)*

## Discussion

This study explored the views of hospital managers on compliance to patient safety culture dimensions in selected hospitals across three regions in Ghana.

The findings indicate that the managers experienced both positive and negative practices in patient safety culture. For example, open communication about patient safety, teamwork within units, the existence of organisational learning, and managers consulting with subordinates regarding patient safety, were evident in two regions. The study further revealed that good and open communication, as well as the use of various communication methods, including the adoption of WhatsApp social network applications enabled flexible communication within teams. This finding is consistent with a Palestinian study which postulated that the dimension of communication openness was a predictor of the overall perceptions of safety [30]. This is an indication of a promising development of patient safety culture in the study regions, although there are numerous areas that require improvement.

In some regions, managers exhibited good handover and information exchange practices while these were lacking in most health facilities in the other regions. The reasons for sub-standard handover are worrying, albeitthey resonate with the study of McElroy et al. [31]. For example, lack of escorting of patients from OPD and reporting late on duty, are indicators of deteriorated levels of care, demotivation and loss of professional and ethical values. Although participants stated that there is a shortage of staff as suggested by Asamani, et al. [32], the authors are of the view that task-sharing and improved commitment to work among health professionals could improve the current sub-standard handing over processes. Moreover, some participants mentioned that some shortages were artificial and were largely occasioned by staff pursuing further studies without study leave. However, others were on approved study leave, maternity leave as well as annual leave. So it depends on the time of the year. When everyone is at work there is no shortage. Therefore, the Ministry of Health is unable to employ additional staff in the absence of those on leave, only to lay them off when everyone returns to work. The finding on extreme shortage of staff corroborates those of Akologo et al. [27] who found a low positive rating on staffing as a patient safety culture dimension, in three selected hospitals of the Upper East region in Ghana. Being a quantitative study Akologo et al. [27] could not explain the cause of the perceived shortage; hence this current study fills this gap. Studies conducted elsewhere such as Tanjung [33] in Indonesia, and Caldas [34] in Brazil also re-echoes the incessant staff shortages in health facilities. These findings imply that human resource management in hospitals should consider skills building, and repurposing of staff who are not physically fit to be in the clinical environment. We recommend that factors that are within the manager’s control such as proper planning of leave and capacity building, be revisited as a matter of urgency, as such actions can lead to immediate achievements towards patient safety culture interventions. Tanjung et al. [33], assert that the provision of human resources is the most basic immediate and prudent action to improve patient safety.

As suggested by nurses in a study in Iran, managers should be concerned about the psychological, and emotional care of their staff, as their supportive behaviour, inspires the nurses to promote their abilities and apply safety culture measures [35]. Failure to create general safety in hospitals and a reactive management approach negatively impacts the patient safety culture in that setting. As attested to by Farokhzadian, et al. [35] shortage of human and medical resources contributes to this. Management of health facilities should be concerned about absenteeism and presenteeism as this endangers the lives of patients in the end. Since management support is critical in creating a patient safety culture. The lack of support with resources has led to improvising and thus compromisingr patient safety. These findings suggest that management has not prioritised patient safety and in fact exhibits poor commitment to same. Findings on lack of managerial support with resources, although common [18, 36–38], are also in agreement with those of Mawuena [39] who investigated the implications of resource constraints and high workload in Ghanaian hospitals. The study by Mawuena [39] also found health professionals resorting to silence, when they do not experience positive benefit from verbalising their frustration over lack of resources. It is recommended that management prioritizes patient safety by committing more financial, human and logistic resources into health facilities. This when implemented will translate rhetoric into practice.

Our findings on organisational learning resonate with those of Otchi et al. [40], and Zabin, et al. [31] who found that health professionals rated themselves high for organisational learning in Northern and Southern parts of Ghana, and Palestine respectively. Albeit, our study revealed different approaches utilised for organisational learning and the lack of application of the knowledge learnt.. Lack of knowledge application suggests that there is a poor intrinsic motivation to learn, since self-driven learning often leads to the application of knowledge [41].

Several weaknesses regarding reporting of adverse events, including fear to report (due to stigmatization and victimization), selective reporting, lack of mandatory documentation of adverse events, reflect on poor leadership and governance of patient safety culture in these hospitals. The findings suggest that there is a need to normalise just culture in the health establishments, to support the desired patient safety culture principles. The findings are similar to those of Zabin, et al. [31] who suggested that awareness is needed regarding the need to report errors and advocated blaming the process instead of the person.

Additionally, our findings are consistent with other studies [18, 27, 42]; our study confirms the ongoing blame culture but further elaborates on circumstances under which managers choose to blame. A United Kingdom study [43], investigated health professionals’ response to observing their colleagues making errors and found that shouting was often used to disrupt the actions that could lead to adverse events, even though they knew it was not the best response. One major concern from this study’s findings is the inconsistencies in approaches used by managers in response to error reporting. These indicate a lack of standardisation, thereby allowing managers to respond in any manner according to their personalities. Such findings suggest that there is a need for developing standard operating procedures on how to react to reporting of an error, and that standardised training should take place across all regions.

This study’s findings emphasizes the importance of good teamwork within units, an attribute which is corroborated by Muftawu [5], who also found teamwork within units rated high in a teaching hospital in Ghana. A study in Iran, found teamwork to be significantly associated with lower occurrences of adverse events and better adverse events reporting [44]. The findings of this study suggest that teamwork is encouraged within units, but not much have been done to encourage teamwork across units/departments. The reported lack of teamwork across units or departments may compromise patient safety since the key criteria for teamwork is communication. Thus, poor inter-department teamwork may influence how departments communicate with each other. A USA study [31] reported that interdepartmental relationships negatively affected patient-related communication. Other studies elsewhere have also found poor teamwork across units [45, 46] to negatively affect handover and continuity of care. The weaknesses in compliance to patient safety culture dimensions in these hospitals are of major concern, more so when they are reported by hospital managers. Evidence suggests that managers are more lenient in evaluating safety in their hospitals [47, 48] and that they look at culture more positively than other staff. The fact that they could report these challenges indicates that the reported challenges could be the tip of the iceberg.

### Limitations and strengths

The current study’s findings may have been limited by the following: this was a cross-sectional study, meaning the findings reflect the views of participants at the point in time during the interviews and may change overtime. Additionally, given the topic discussed in this study, most participants found this as an avenue to vent their frustrations; this could potentially introduce social desirability bias. However, a trained interviewer conducted the interviews and kept probing the participants to cite examples of what they observed in their hospitals. Being qualitative, the results cannot be generalised, however, critical lessons can be learnt from the findings. Nonetheless, there are several strengths from this study; patient safety culture is often investigated using quantitative approaches, leading to an abstract view of the findings. A 20-year-long scoping review done in 2021 [21], on methodologies used to investigate patient safety culture, found that quantitative approaches are predominant with the authors recommending a need for more qualitative approaches, to explore the reasons behind the low and high ratings. To the best of our knowledge, our study is one of the first studies to apply the patient safety dimensions framework, in a qualitative approach to exploring the views of hospital managers in the Ghanaian context. The approach allowed us to unpack the nuances of contextual challenges constraining patient safety culture in the hospitals under study. For example, we unveiled more reasons behind the usual rating of patient safety culture, such as contributors to the shortage, perceived lack of implementation of organisational learning, various approaches in response to errors, and contributors to poor handover. For future studies, we recommend a mixed method approach so that survey findings can be compared alongside qualitative results in the same setting.

## Conclusion

There is a need to normalise just culture in the hospitals, and to train managers on effective reporting of errors. There is also the need to standardise such approaches, to prevent managers from exploitatingsubordinates as every manager currently responds according to his preferred style. Management support with resources, motivation of personnel and with training on patient safety is urgent, to create a positive patient safety culture.

### Implications for managers or decision-makers

Strong leadership, management and governance are required to monitor and discipline staff absenteeism as well as reporting late on duty. There is a need to set standardised policies ensuring that handover is not delegated to student nurses. Human resource management should further include education regarding the link between handover and patient safety. The executive management of health facilities could be more consistent and not only be supportive when accreditation is due. Additionally, management should provide necessary resources for use by health professionals to prevent cutting corners and regular improvision. This qualitative study as permitted frontline managers to air their views. It is hoped that executive or top managers will take a cue from the study findings to correct perceived wrongs. Therefore, policymakers and managers could reflect on these findings and apply recommendations from this study to further improve patient safety in health facilities. The study’s findings are particularly relevant for other developing countries with similar healthcare systems.

## Supplementary Information

**Additional file 1: Table S1.** Demographic data of participants.

**Additional file 2: Table S2.** Interview guide.

## Data Availability

The datasets used and/or analysed during the current study are available from the corresponding author on reasonable request.

## Abbreviations

AAA: Aaron Asibi Abuosi
DN: Delight Nyonator
FGD: Focus Group Discussion
GHS: Ghana Health Service
IST: Immaculate Sabelile Tenza
OPD: Outpatient Department
PYAA: Priscilla Yeye Adumoah Attafuah
PSC: Patient Safety Culture
WHO: World Health Organization
