# Supplementary material for: Hospital managers’ views on the state of patient safety culture across three regions in Ghana
Source: BMC Health Serv Res. 2022 Oct 29;22:1300. doi: 10.1186/s12913-022-08701-z (PMC9617533; doi:10.1186/s12913-022-08701-z)
Supplement: Supplementary file 1 — Additional file 1: Table S1. Demographic data of participants. [file 12913_2022_8701_MOESM1_ESM.docx]

**Additional file 1 Table S1 Demographic data (N = 114)**

| **Variable** | **Category** | **Frequency**  **(n)** | **Percentage**  **(%)** |
| --- | --- | --- | --- |
| **Zone/Region** | Upper East Region | 24 | 21.1 |
|  | Bono Region | 33 | 28.9 |
|  | Greater Accra Region | 57 | 50.0 |
|  |  |  |  |
| **Gender** | Female | 39 | 34.2 |
|  | Male | 75 | 65.8 |
|  |  |  |  |
| **Age group** | 35 – 40 | 48 | 42.1 |
|  | 41 – 45 | 33 | 29.9 |
|  | 46 – 50 | 19 | 16.7 |
|  | Above 50 | 14 | 12.3 |
|  |  |  |  |
| **Profession** | Nurse managers | 70 | 61.4 |
|  | Pharmacy managers | 10 | 8.8 |
|  | Accounts managers | 12 | 10.5 |
|  | Janitorial heads | 2 | 1.7 |
|  | Quality assurance Managers | 4 | 3.5 |
|  | Laboratory heads | 6 | 5.3 |
|  | Administrator Managers | 10 | 8.8 |
