# Supplementary material for: Hospital managers’ views on the state of patient safety culture across three regions in Ghana
Source: BMC Health Serv Res. 2022 Oct 29;22:1300. doi: 10.1186/s12913-022-08701-z (PMC9617533; doi:10.1186/s12913-022-08701-z)
Supplement: Supplementary file 2 — Additional file 2: Table S2. Interview guide. [file 12913_2022_8701_MOESM2_ESM.docx]

| **Additional File 2 Table S2 Interview guide**  **PATIENT SAFETY CULTURE PROJECT**  **Interview Guide for Managers** |  |
| --- | --- |
| **Focus group discussion agenda items** | |

1. **Welcome and introduction of researchers, participants, and the purpose of the focus group discussion**
2. **Explanation of study information sheet**
3. **Brief explanation of study terms like patient safety and patient safety event, and patient safety culture dimensions**
4. **Signing of consent forms and confidentiality agreement**
5. **Discussion of basic rules to guide the discussion, e.g respect for other opinions, and being free to talk.**

| ***Study items defined:***   - ***“Patient safety”*** *is defined as the avoidance and prevention of patient injuries or adverse events resulting from the processes of healthcare delivery.* - ***A “patient safety event”*** *is defined as any type of healthcare-related error, mistake, or incident, regardless of whether or not it results in patient harm.* - ***Patient safety culture dimensions:*** *The dimensions include Communication about Error, Communication Openness, Handoffs and Information Exchange, Hospital Management Support for Patient Safety, Organizational Learning—Continuous Improvement, Reporting Patient Safety Events, Response to Error, Staffing and Work Pace, Supervisor, Manager, or Clinical Leader Support for Patient Safety and Teamwork* |
| --- |

1. **Completion of demographic data by participants**
2. **Open discussion focused on each of the dimensions of patient safety culture**
3. **Closure**

SECTION 1: DEMOGRAPHIC DATA

1. What is your gender? Male 🞎 Female 🞎
2. What is your age in years? …………..
3. What is your position in this hospital?

Select ONE answer.

| **Nursing**  🞎1 Nurse Manager (Matron)  🞎2 Unit Head (e.g. Ward or OPD In-charge)  🞎3 Other, specify……………………………..  **Medical**  🞎4 Medical Director  🞎5 Unit Head (e.g. Dental or Eye Unit In-charge)  🞎6 Other, specify……………………………..  **Other Clinical Position**  🞎7 Dietitian In-Charge  🞎8 Pharmacist, Pharmacy Technician In-Charge  🞎9 Physical, Occupational, or Speech Therapist In-Charge  🞎10 Psychologist In-Charge  🞎11 Social Worker In-Charge  🞎12 Technologist, Technician (e.g., MRI, Lab, Radiology) | **Support**  🞎13 Facilities In-Charge  🞎14 Food Services In-Charge  🞎15 Housekeeping, Environmental Services In-Charge  🞎16 Information Technology, Health Information Services, Clinical Informatics In-Charge  🞎17 Security In-Charge  🞎18 Transportation In-Charge  🞎19 Unit Secretary, Receptionist, Office Staff In-Charge  **Other**  🞎20 Other, please specify: |
| --- | --- |
|  |  |

What is your primary unit or work area in this hospital?.............................................

*For Office use Only: Participant code: …………………Region…………….Hospital…………….*

**SECTION 2: OPEN DISCUSSION FOCUSED ON EACH OF THE DIMENSIONS OF PATIENT SAFETY CULTURE**

**Main opening question:** To what extent are the patient safety culture dimensions practiced in your units?

1. **Let’s talk about Teamwork**

**Probes:**

- How would you say teamwork is practiced or not practiced in your unit?
- Give examples
- What could be the contributing reasons

1. **Let’s talk about staffing and work Pace**

**Probes:**

- What is the staffing situation with regards to workload?
- Describe staff working hours whether they are appropriate and if staff feel rushed.
- Describe the temporary staff or part-time staff situation of the hospital?
- What contributes to this?

1. **Let’s talk about organizational learning—continuous improvement**

**Probes:**

- Tell us about your observation of organizational learning in relation to patient safety in this hospital?
- How is it practiced?
- How is the learning implemented?

1. **Let’s talk about response to error**

**Probes:**

- In your observation, what happens to a staff who makes a mistake or commits an error?
- What is the standard approach to response to error?
- Would you like to share some examples?

1. **Let’s talk about supervisor, manager, or clinical leader support for patient safety**

**Probes:** Describe what supervisors, managers, or clinical leaders do under the following circumstances with regards to staff involvement.

- When suggestions are needed to improve patient safety.
- When work needs to be done faster during busy times, even if it means taking shortcuts
- What actions are taken to address patient safety concerns brought to the attention of supervisors, managers, or clinical leaders?

1. **Let’s talk about communication about error**
   1. Could you share how your unit communicate about an error that has happened?
   2. How is the staff involved in error prevention or any changes in response to the error?
   3. Could you share some examples?
2. **Let’s talk about communication openness:**

**Probes:**

- How is the staff in your units feel about communicating if they see something that may negatively affect patient care?
- How is your staff feel about communicating to someone with more authority when they see him doing something unsafe for patients?

1. **Let’s talk about reporting patient safety events**

**Probes:**

- When a mistake is caught and corrected before reaching the patient, how is this reported?
- When a mistake reaches the patient and could have harmed the patient, but did not, how is this reported?
- Could you share examples?

1. **Let’s talk about hospital management support for patient safety**

**Probes:**

- How does hospital management show that patient safety is a top priority?
- Your experience in provision of resources?
- What contributes to this?

1. **Let’s talk about handoffs and information exchange**

**Probes:**

- Can you describe how important patient care information is transferred across hospital units and during shift changes in this unit/work area?
- Could you share some examples?

**Closure:**
